# Resource landscape, microbial activity, and community composition under wintering crane activities in the Demilitarized Zone, South Korea

**DOI:** 10.1371/journal.pone.0268461

**Published:** 2022-05-13

**Authors:** Kyungjin Min, Myung-Ae Choi

**Affiliations:** Center for Anthropocene Studies, Korea Advanced Institute of Science and Technology, Daejeon, South Korea; The Ohio State University, UNITED STATES

## Abstract

Endangered cranes migrate to the Demilitarized Zone in Korea in search for habitat and food during winter. While cranes have the potential to influence soil biogeochemical processes via dropping, foraging, and walking, few studies have investigated ecological roles of migrating birds in the new habitat. Here, we explored how cranes alter resource landscape (the amount and quality of carbon) and microbial community in soil. We set up control (fenced, no crane access) and treatment (free crane activities) plots (n = 6, respectively) in a rice paddy, and collected soils at 0–15 cm three months after the crane migration. Soils were tested for total carbon, total nitrogen, water extractable organic carbon, and Diffuse Reflectance Infrared Fourier Transform Spectroscopy, along with microbial parameters (biomass, respiration, community composition). The wintering crane activity significantly increased total carbon and nitrogen contents, but decreased the ratio of CH (aliphatic) to COO (carboxylic) in soil. Also, both microbial biomass and respiration was greater in soils under crane activities. Bacterial and fungal community composition differed with or without crane activities, with treatment soils harboring more diverse microbial communities. Our results demonstrate that crane migration created a distinct system with altered resource landscape and microbial community, highlighting beneficial effects of migratory cranes on the soil biogeochemical processes in rice paddies. This study may help encourage more farmers, local governments, and the public to participate in crane conservation campaigns targeted at rice fields.

## Introduction

Habitat loss, excessive hunting, and poisoning have decreased the number of cranes, leading to 11 out of 15 crane species threatened with extinction under the International Union for Conservation of Nature (IUCN) Red List Categories system [1]. Once common wintering birds in the Korean Peninsula [2], cranes are now observed in few areas including the Demilitarized Zone (DMZ) and Civilian Control Zone (CCZ) in South Korea. Previous studies have documented the crane migratory pathways [3, 4], population change [5, 6], and foraging behavior [7]. In spite of these efforts, however, we do not know the effects of crane migration on the biogeochemical processes in the new habitat. Understanding the direction and magnitude of the changes in biogeochemical processes after the crane migration would help us better assess ecosystem functions during winter, a critical step to evaluate beneficial effects of cranes and to obtain a momentum for the crane conservation efforts among local farmers, governments, and the public.

Cranes may alter biogeochemical processes of the new habitat by recharging, redistributing, and refreshing resources during winter. Bird fecal matter is rich in carbon (C), nitrogen (N), phosphorus, and potassium [8–10], likely serving as fertilizer. As such, ecosystems with limited resource availability can be influenced by the input of bird fecal matter. For example, Firth et al. [10] demonstrated that winter migration of goose and duck into rice fields increased total C and N concentration in soil, with a potential to replace 8.2–27.5% of the fertilizer recommendation dose. Also, cranes may redistribute resources within and across ecosystems. Because cranes are omnivores feeding on fish, insects, snails, and grains [1], they may transfer nutrients across ecosystems by taking up relatively N-rich fish or aquatic invertebrates from streams or reservoirs and defecating in other places. Using ^15^δN, Kameda et al. [11] found that great cormorant (*Phalacrocorax carbo*) transports N from fresh water to forest ecosystems via droppings and collection of N-rich nest material. Alternatively, birds may refresh the quality of resources via digestion or foraging. Bird fecal matter is partly or completed digested, so it may provide readily available resources to other organisms [10]. Or, foraging itself can alter the amount, chemical composition, and physical condition of rice straw left in the field after harvest [12].

Changes in the resource quantity and quality often alter soil microbial activity and community composition, with no clear directional changes [13–16]. A meta-analysis showed that microbial biomass and respiration decreased with N additions in all ecosystems [17]. Likewise, N additions decreased a suite of microbial extracellular enzyme activity that breaks down soil organic matter [18, 19] and led to less diverse fungal communities [20]. Using modeling and meta-analysis approaches, Whittinghill et al. [21] demonstrated that decreases in the rate of microbial activity under N additions were due to increases in less bioavailable, slow-cycling C pool. Alternatively, increases in the resources can enhance soil organic matter decomposition. Soil microbial growth and respiration in rhizosphere are often higher than those in bulk soils, due to root exudates that contain readily-available resources to microbes [22, 23]. Microbial diversity was higher at surface soil than microbial diversity at 2 m of soil depth due to greater C availability [24]. High N availability led to microbial communities with more bacteriodetes and proteobacteria, and less acidobacteria [25]. Because soil microbial communities mediate biogeochemical processes [26, 27], any changes in their activity and community composition are likely to affect nutrient cycling in agroecosystems in the DMZ.

Our study site is Cheorwon, Kangwon Province, South Korea. Red-crowned cranes (*Grus japonensis*) and White-naped cranes (*Grus vipio*) and 5 other crane species come to Cheorwon in mid-October, and leave early March for north-eastern China and Russia for breeding [2]. Both Red-crowned cranes and White-naped cranes are endangered species, classified as Vulnerable at the IUCN’s Red List [28, 29]. Cranes were observed in Cheorwon in the 1970s for the first time since the Korean War (1950–1953), and their population started to pick up in the mid-1990s. The number of Red-crowned cranes grew from 372 in 1999 to 1,126 in 2021, while White-naped cranes showed a 10-fold increase from 474 to 5,330 during the same period [30]. Since the early 2000s, conservationists, environmental authorities, and local farmers started to take actions to protect cranes during winter. These practices include creating roosting places by flooding rice paddy fields, in addition to securing food sources by keeping rice straw in field after harvest, sprinkling out grains, and leaving the fields unplowed. By doing so, they hope to protect the endangered cranes, and to promote eco-tourism and the brand value of the rice they produce.

Our goal was to assess the effects of migratory wintering cranes on the biogeochemical processes in rice paddy agroecosystems around the DMZ [31]. We hypothesized that crane activities will increase the quantity and quality of resources in soil, and that microbial biomass, respiration, and diversity will be higher under crane activities. To test these, we collected soils at 0–15 cm three months after the crane migration from treatment (free crane activities) and control (fenced, no crane access) plots (n = 6, respectively). We assessed microbial activity (basal respiration), biomass (DNA content, substrate-induced respiration), and microbial community composition (fungi and bacteria), along with soil chemistry (total C, N, water extractable organic carbon (WEOC), Diffuse Reflectance Infrared Fourier Transform Spectroscopy (DRIFT)). To our knowledge, this is the first attempt to consider wintering cranes as biogeochemical modifiers in DMZ. By examining the impact of crane activities on agricultural soils, the results of this study can attract interests from researchers, farmers, and general public in conserving the endangered cranes.

## Materials and methods

### Study site

The study site is in Cheorwon, nested within the DMZ and CCZ areas (38°16’42.9"N, 127°12’48.3"E). The mean annual temperature is 10.2°C, with a mean daily maximum of 16.2°C and mean daily minimum of 4.7°C. The mean annual precipitation is 1391.2 mm [32]. Parent material in the area is basalt and the soils are classified as Fine, mesic family of Aeric Epiqauults (Dongsong Series) [33].

Cranes make use of the northern part of Cheorwon, approximately 10,000 ha including the rice fields in and around the CCZ, Hantan River, and the wetlands inside of the DMZ [34]. A total of 30 ha rice fields, spanning over three sets of rice paddies, were flooded after harvest in 2020 to provide a winter habitat for the cranes [35]. In addition, rice straws were kept in a total of 800 ha rice fields as a way of keeping waste grains within the rice paddies for the cranes to forage [36]. The average percentage of grain loss by combine harvesters is 3% in Cheorwon, producing over 2,000 t of waste grains every year (personal communication with Seo, K). Waste grains are the primary food source to the cranes, while fish and aquatic invertebrates in the farm waterways are also consumed [1]. Given that an adult crane consumes 200–250 g of grains (corn, rice, beans, 4% of body weight) per day, one crane need at least 37.5 kg of grains over the winter (120 days) [37]. Local farmers and conservationists sprinkle grains in the fields later in the winter when the waste grains are running out.

The farmers allowed us to use one rice paddy among the flooded ones for this study (38°16’55.27"N, 127°12’47.56"E). According to the farmers, the majority of the cranes roost in the flooded rice fields at the early phase of their migration until mid-Dec when the wetlands inside of the DMZ are frozen and ready for cranes to roost in. In this regard, the selected study site may offer a useful window to examine the effect of crane activities to the soil. After rice was harvested in September, 2020, a 3 m x 3 m of levee was established at the corner of the field (45 m x 10 m) and the area was covered with an anti-bird net to block the access of cranes (control). The rest of the field served as treatments, where cranes can come for food and shelter. Soil samples were collected at 0–15 cm from the control and the treatment (n = 6, respectively) with a hand auger (diameter = 38 mm, Eijkelkamp, Netherlands), before (October, 2020) and after (January, 2021) the migration of wintering cranes. Soils were homogenized with hand, and roots and rocks were picked manually. Before the migration, soil pH, gravimetric water content, and WEOC was 6.14 ± 0.08 (mean ± standard error), 33.21 ± 1.29 (%), and 51.47 ± 3.86 (μg g^-1^ dry soil), respectively. Along with soil samples, crane droppings were collected from the treatment plots. All samples were kept in a cooler, sent to Korea Advanced Institute of Science and Technology, and stored at 4°C before chemical and biological analyses.

### Crane monitoring–*in situ* surveys and trail cameras

**Fig 1:**
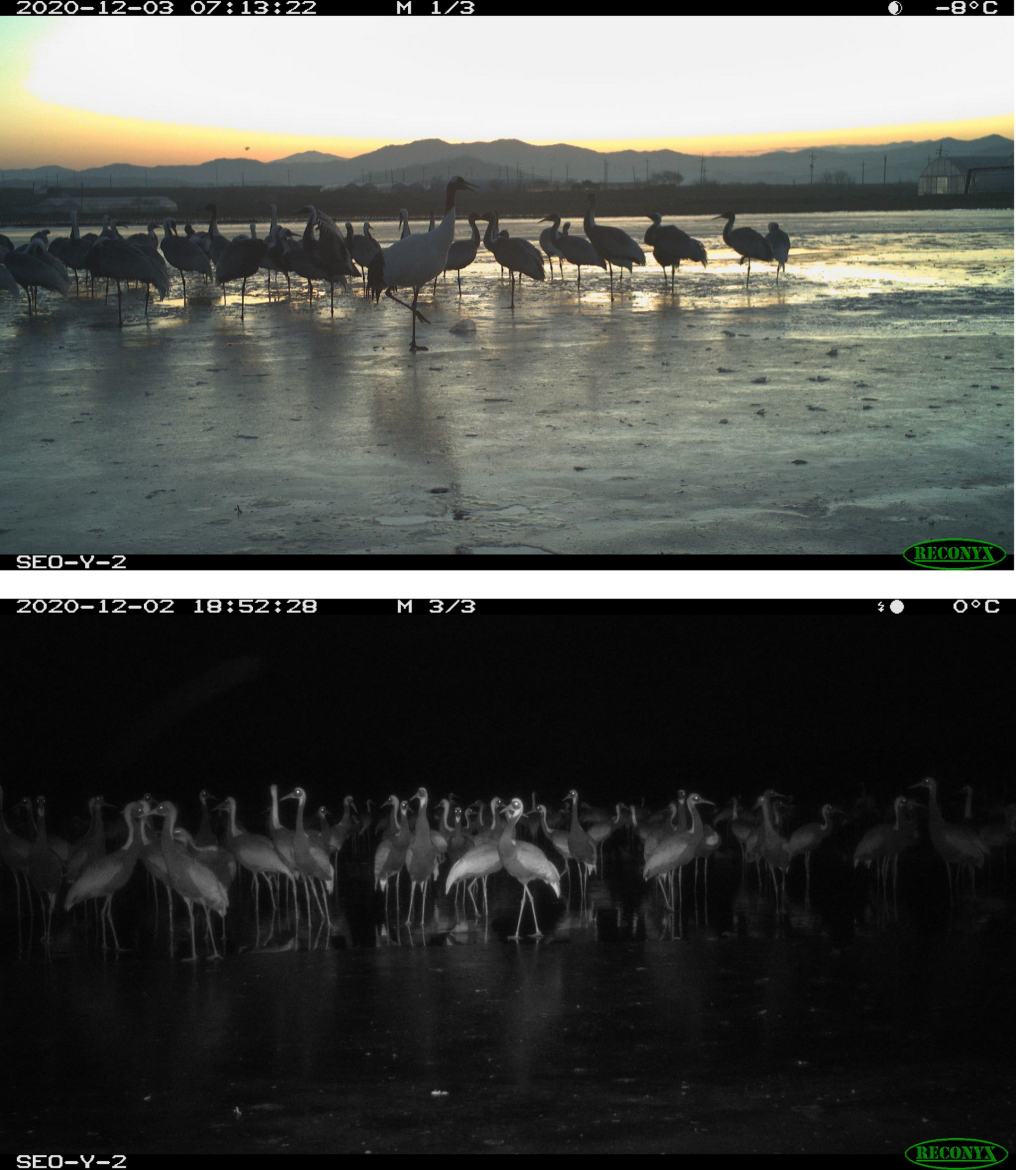
Example picture from a trail camera (Reconyx HF2X, Reconyx, USA) in the treatment plot (flooded, but not fenced) for red-crowned cranes (*G*.*japonensis*).

Providing the exact number of how many of them used the study site is challenging because of the technical difficulties of counting large flocks of birds, let alone their mobile nature [38]. In spite of the difficulties, we strived to quantify the number of cranes in the study site. First, we employed *in situ* surveys to estimate overall number of cranes migrating in Cheorwon region. One of the authors participated in monthly crane census over the winter of 2020–2021. In addition to the surveys, we also use cameras, artificial intelligence, and volunteer citizens to monitor cranes. We set up three trail cameras adjacent to the rice field, one for the control plot (Browning BTC-8A, Blucoil, USA) and two for the treatment plots (Reconyx HF2X, Reconyx, USA), to monitor the presence of cranes between October, 2020 and March, 2021 (Fig 1). To accelerate the analysis process, we have been developing artificial intelligence (AI) algorithm to identify and count crane species [39]. We have kept training the AI with large sets of trail cam images and will improve the performance with the help from volunteer citizens to accurately record the number of crane populations [40].

### Soil physicochemical properties

Total C and N content of soil was measured on 105°C dried, ground soil samples with an Elemental Analyzer (FlashEA 1112, Thermo Finnigan, Italy). We quantified WEOC concentration by mixing 7 g of fresh soil with 35 mL of deionized water, shaking the mixture on an orbital shaker at 200 rpm for 4 h, filtering the mixture through a 0.45 μm filter [41] (Sterile Minisart 16555K, Sartorius, Germany), and determining total organic C content in the filtrates using Shimadzu TOC-L (Shimadzu, Japan). Dry samples were mixed with KBr (1:10, w/w) and reflectance was measured between 4,000 and 400 cm^-1^ averaged over 32 scans for Diffuse Reflectance Infrared Fourier Transform Spectroscopy [42] (DRIFTS; Nicolet iS50, Thermo Fisher Scientific, USA). We calculated the ratio of aliphatic (CH, between 2,976 and 2,898 cm^-1^ and between 2,870 and 2,839 cm^-1^) over carboxylate (COO, between 1,450 and 1,360- cm^-1^) residues to estimate the quality of organic C. The lower, the more processed and more likely resistant to further decomposition [42, 43]. All the analyses were performed at Korea Advanced Institute of Science and Technology Analysis Center for Research Advancement, except for WEOC that was examined at Korea Institute of Geoscience and Mineral Resources.

### Basal respiration and substrate-induced respiration

For basal respiration and substrate-induced respiration, we used methods described in Min et al. [41]. Briefly, a 20 g of fresh soil was weighed in a 530 mL jar and kept at 22°C overnight (12 jars = 2 treatments x 6 replicates). Jars were sealed with a lid equipped with a Non Dispersive Infrared CO_2_ sensor (K30, CO_2_ Meter, USA). CO_2_ concentration in the jar was recorded every 10 s for 10 min to generate a positive slope between CO_2_ concentration and time. The slope was used to calculate respiration rate at a given time (μg C-CO_2_ h^-1^ g^-1^ dry soil). We repeated measuring respiration rate every 30 min for 2 h (thus, four times). We refer this as basal respiration rate before adding substrates or adjusting water content and presented mean basal respiration [41] (n = 4). After measuring basal respiration rate, we added 40 mg of yeast powder extract per g soil, adjusted soil water content at 60% of water holding capacity, stirred the mixture, and monitored respiration rate every 30 min for 24 h. Microbial respiration rate after the yeast extract addition is called substrate-induced respiration here after [41, 44, 45].

### Microbial community analysis

We extracted soil microbial DNA following the manufacturer’s instructions from the DNeasy Power Soil Kit (Qiagen, USA). DNA extracts were stored at -80°C and sent to Chunlab Inc. (Seoul, South Korea [46] on dry ice for bacterial and fungal analyses. PCR amplification was performed using fusion primers targeting from V3 to V4 regions of the 16S rRNA gene (bacteria) and ITS (fungi) (further information in S1 and S2 Files). For bacteria, the EzBioCloud 16S rRNA database [47] was used for taxonomic assignment and chimeric reads were filtered on reads with <97% similarity by reference. For fungi, we used unite database [48]. The alpha diversity indices (Shannon and OTUs) were estimated and compared between the control and treatment soils using Wilcoxon rank-sum test. We calculated UniFrac and Bray-curtis beta diversity distances and used principal coordinates analysis to visualize the sample differences. All the analytics mentioned above were performed in EzBioCloud 16S-based MTP and unite ITS based MTP, which are a ChunLab’s bioinformatics cloud platform [49].

### Statistical analyses

All the data except for microbial community data (see above) was checked for normal distribution (Shapiro-Wilk test) and has met the requirement for parametric analysis. We used a one-way ANOVA to test the effects of crane activities on soil chemical and biological properties. Independent variable was the existence of cranes and response variables were total C, total N, WEOC, the ratio of CH over COO (FTIR), DNA content, substrate-induced respiration, and basal respiration. We used R 4.0.2 [50] and a statistical significance was tested at α = 0.05.

## Results

### The number of cranes

Local farmers’ practices to flood the rice fields after harvest and to keep the rice straws within the rice fields have successfully attracted migrating cranes in the study site. Monthly crane surveys during 2020–2021 revealed that about 1,000 cranes (124 *G*.*japonensis* and 875 *G*.*vipio* in December 2020; 103 *G*.*japonensis* and 824 *G*.*vipio* in February 2021) were counted during the day in the broader rice fields including the study site. The number of the cranes roosting in the study site appears bigger: the census team observed at least 2,154 *G*.*vipio* on 15 Nov 2020, while local farmers claim 3,000–4,500 cranes staying overnight.

Trail cameras generated a total of 22,511 pictures and 1,762 videos of wintering crane and others in the treatment plots at the study site (Fig 1). No cranes were found from 16,859 pictures in the control plot during the experimental period. Crane monitoring is an ongoing project, and the exact number and species have been being analyzed with the help of AI and the volunteer citizens (see Materials and Methods).

### Crane droppings and resource landscape after the crane migration

**Fig 2:**
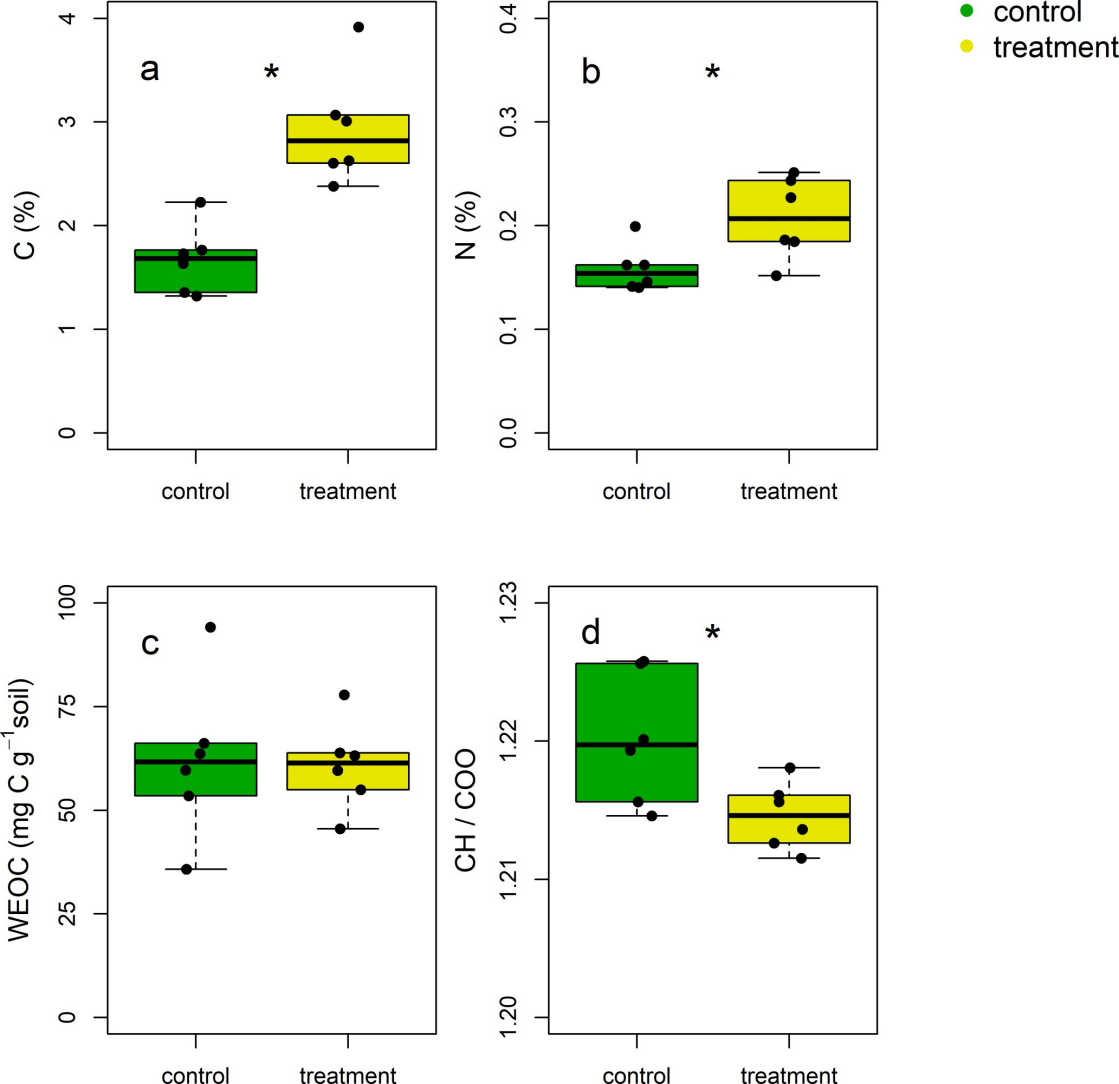
Total (a) C, (b) N, (c) WEOC, and (d) CH/COO of soils three months after the crane migration. Green for control and yellow for treatment soils. An asterisk was put when the difference between the control and treatment soils was significant at α = 0.05 (n = 6).

Crane droppings exhibited significantly different chemical properties from initial soil (Table 1). Total C and N contents of crane droppings were 18 times and 13 times greater than that of initial soil, respectively (p<0.001, respectively). Hence, the input of crane droppings rich in C and N provided a pulse of resources into the soil system, resulting in significant changes in total C, N, and CH/COO ratio after the crane migration (Fig 2; p<0.001, 0.024, and 0.028 respectively). Total C and N contents were greater in soils under crane activities (treatment soils) than in the control soils (Fig 2A and 2B). On average, total C and N increased by 76 and 31% respectively in the treatment soils compared to the control. Contrary to the changes in total C and N, the WEOC content was similar between the control and treatment soils, with an average of 61.5 μg g^-1^ dry soil (Fig 2C). There was no significant relationship between the total C and N contents and the WEOC content (Table 2). The ratio of CH over COO was smaller in the treatment soils than in the control soils (Fig 2D), indicating less plant-derived, but more processed C under crane activities.

**Table 1:** Total C, N, and ratio of CH to COO of initial soil and crane droppings (mean ± 1SD).

	Total C (%)	Total N (%)	CH/COO
Initial soil	1.61 ± 0.19	0.18 ± 0.03	NA
Crane dropping	29.78 ± 3.76	2.42±0.77	1.21±0.0008

*n = 12 and 3 for initial soil and crane droppings, respectively.

**Table 2:** Pearson’s correlation coefficients between final soil conditions (three months after the crane migration) and microbial properties.

	Total C	Total N	WEOC	CH/COO	DNA	SIR	respiration
Total C		**0.88**	-0.06	**-0.60**	**0.84**	**0.81**	**0.78**
Total N			0.13	-0.42	**0.62**	**0.62**	**0.77**
WEOC				0.53	**-0.15**	-0.12	0.30
CH/COO					**-0.74**	**-0.68**	-0.35
DNA						**0.89**	**0.80**
SIR							**0.67**
respiration							

*bold when p<0.05.

### Microbial biomass and respiration

**Fig 3:**
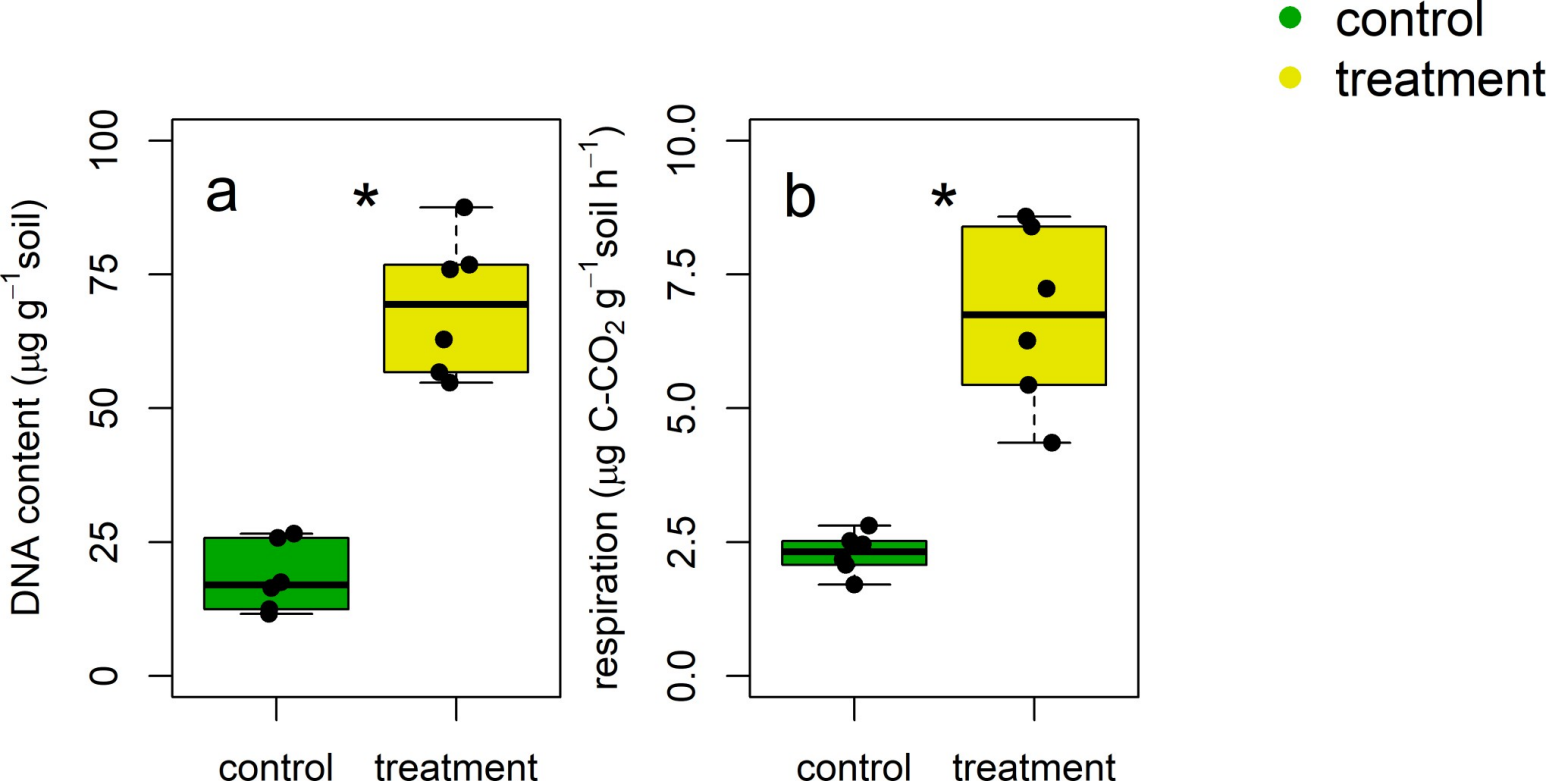
**Microbial biomass for the control (green; no crane access) and treatment (yellow; free crane activities) soils.** We used both (a) static (DNA content) and (b) dynamic (substrate-induced respiration) approaches to determine the size of soil microbial biomass. An asterisk was put when the difference between the control and treatment soils was significant at α = 0.05 (n = 6).

We assessed microbial biomass using static (DNA content) and dynamic (substrate-induced respiration) approaches (Fig 3) after the crane migration. Both results were congruent each other (Pearson correlation coefficient r = 0.89, p<0.001, Table 2) and demonstrated that the crane migration significantly increased microbial biomass (p<0.001 for both approaches). Overall, soils under crane activities (treatment) harbored ca. three times more microbial biomass compared to soils without crane activities (control). Microbial biomass increased with soil C and N contents, but decreased with CH/COO ratio (Table 2).

**Fig 4:**
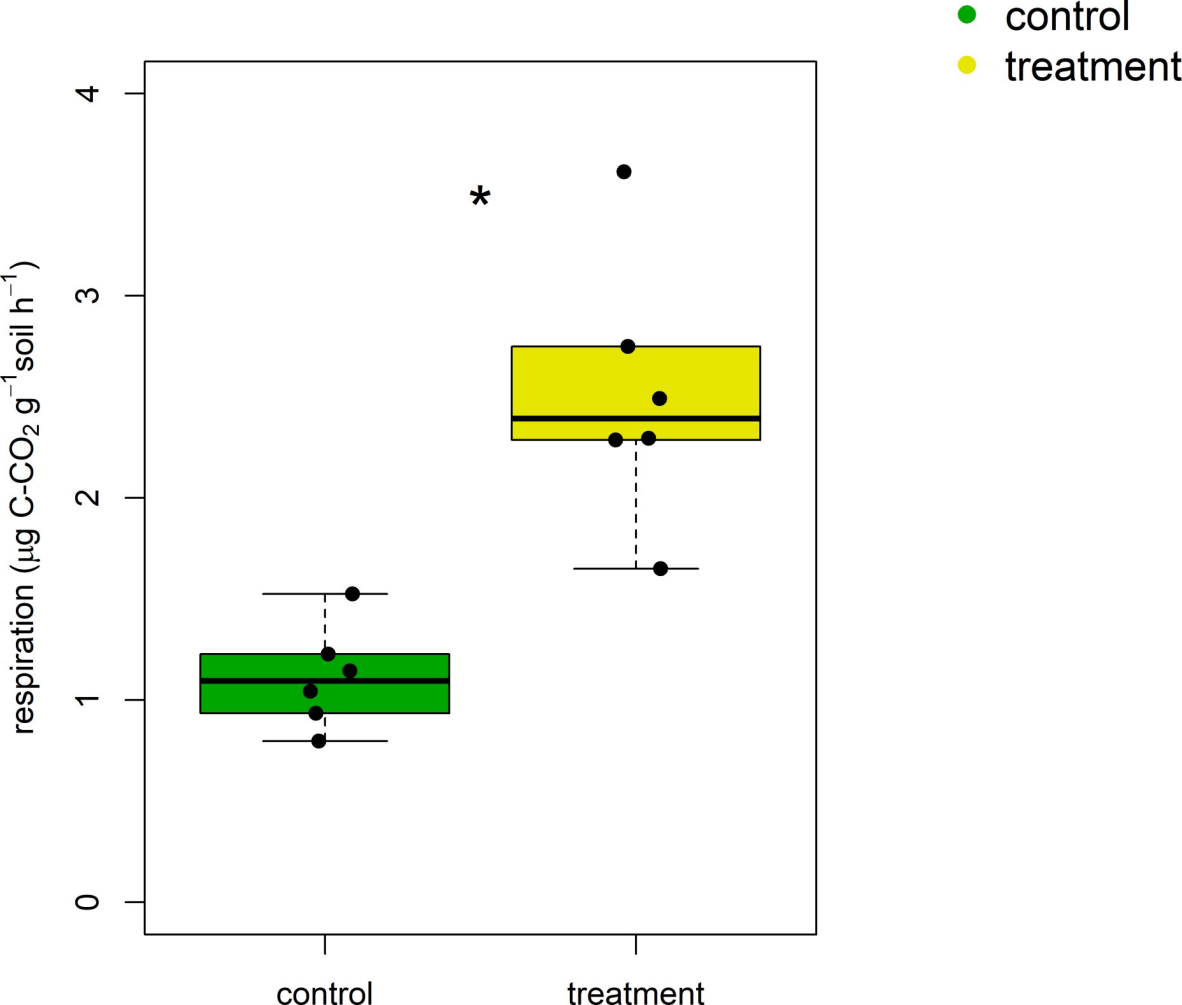
**Microbial basal respiration for the control (green; no crane access) and treatment (yellow; free crane activities) soils.** An asterisk was put when the difference between the control and treatment soils was significant at α = 0.05 (n = 6).

Crane activities not only increased soil microbial biomass, but also enhanced basal respiration rate (Fig 4). On average, respiratory activities in the treatment soils were greater than those in the control soils by 120%. There was a significant, positive relationship between microbial biomass (DNA and SIR) and basal respiration (Table 2).

### Microbial community composition

**Fig 5:**
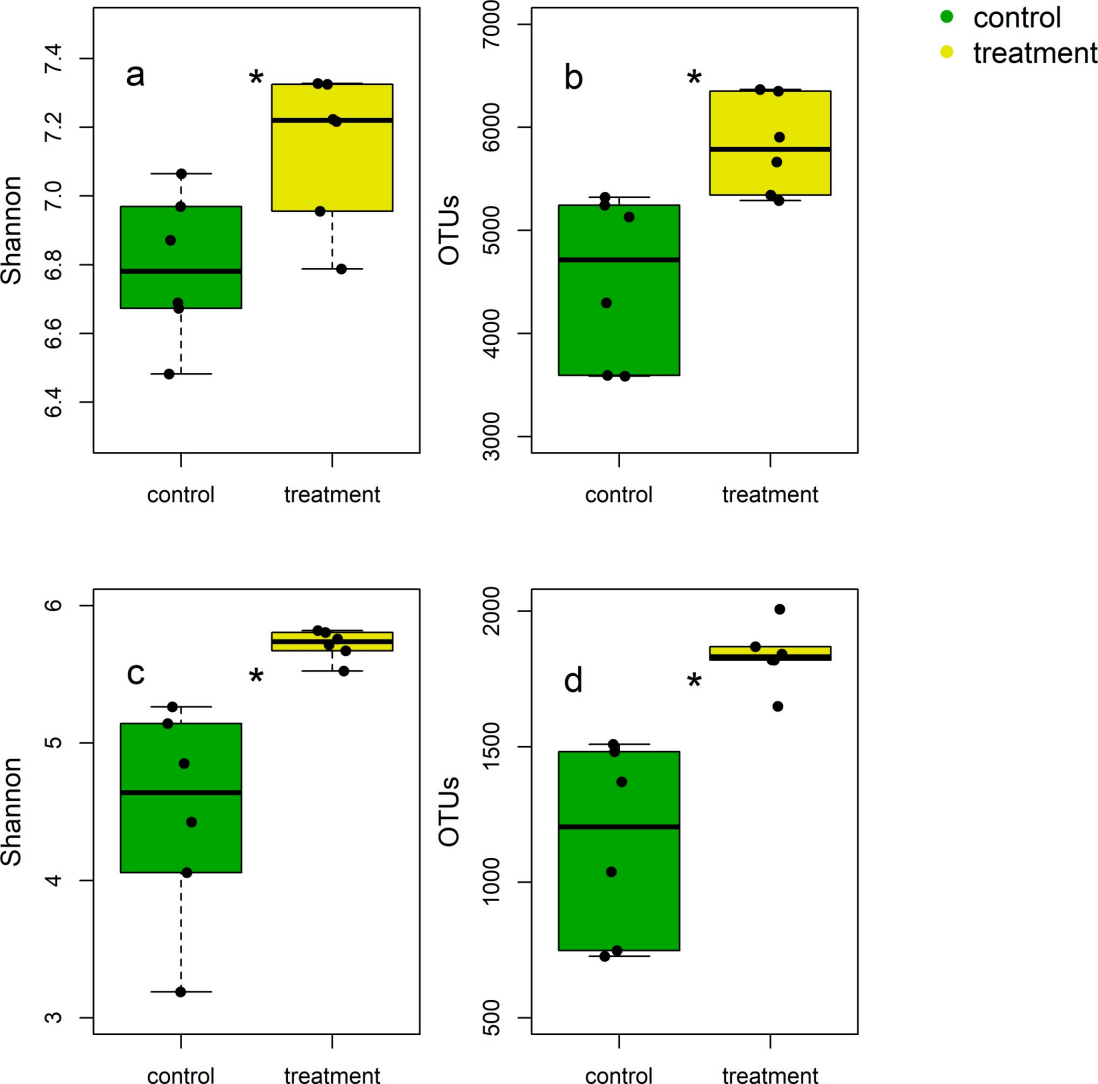
**Microbial alpha diversity in the control (green; no crane access) and treatment (yellow; free crane activities) soils.** Shannon index for bacteria (a) and fungi (c), OTUs for bacteria (b) and fungi (d). An asterisk was put when the difference between the control and treatment soils was significant at α = 0.05 (n = 6).

Soils under crane activities harbored more diverse microbial communities. We tested two Alpha diversity indices, Shannon index and the number of OTUs (Fig 5). Shannon index indicated that the number of species and evenness were great in the treatment soils for both bacteria and fungi, compared to the control soils. The number of OTUs also showed a similar pattern, with greater values for the treatment soils.

**Fig 6:**
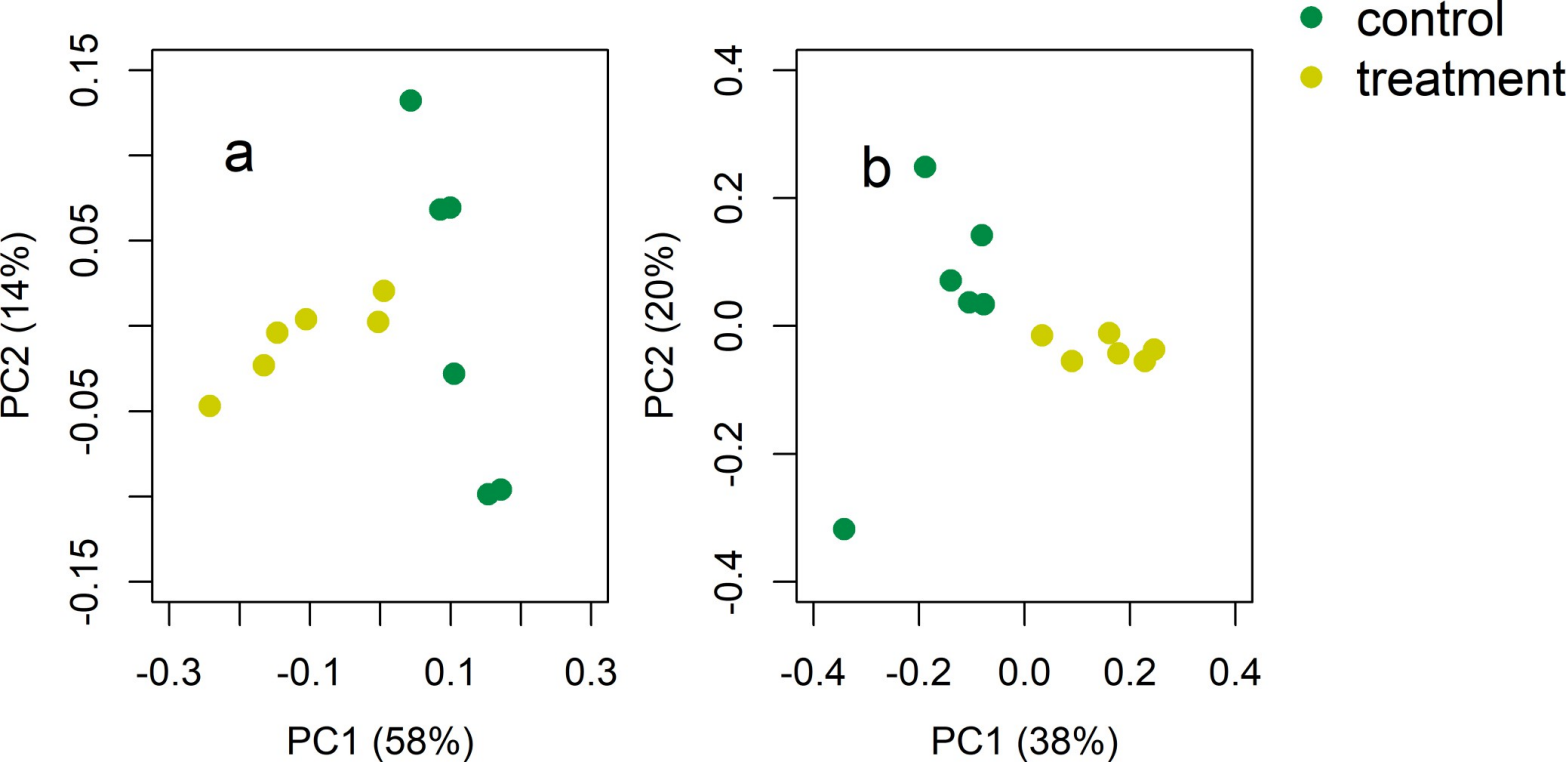
Principal coordinates analysis of the distance among microbial communities. Green symbols are for the control (no crane access) and yellow dots for the treatment soils (free crane activities). For (a) bacteria, Unifrac distance was used, while Bray-Curtis dissimilarity index was used for (b) fungi (n = 6).

**Fig 7:**
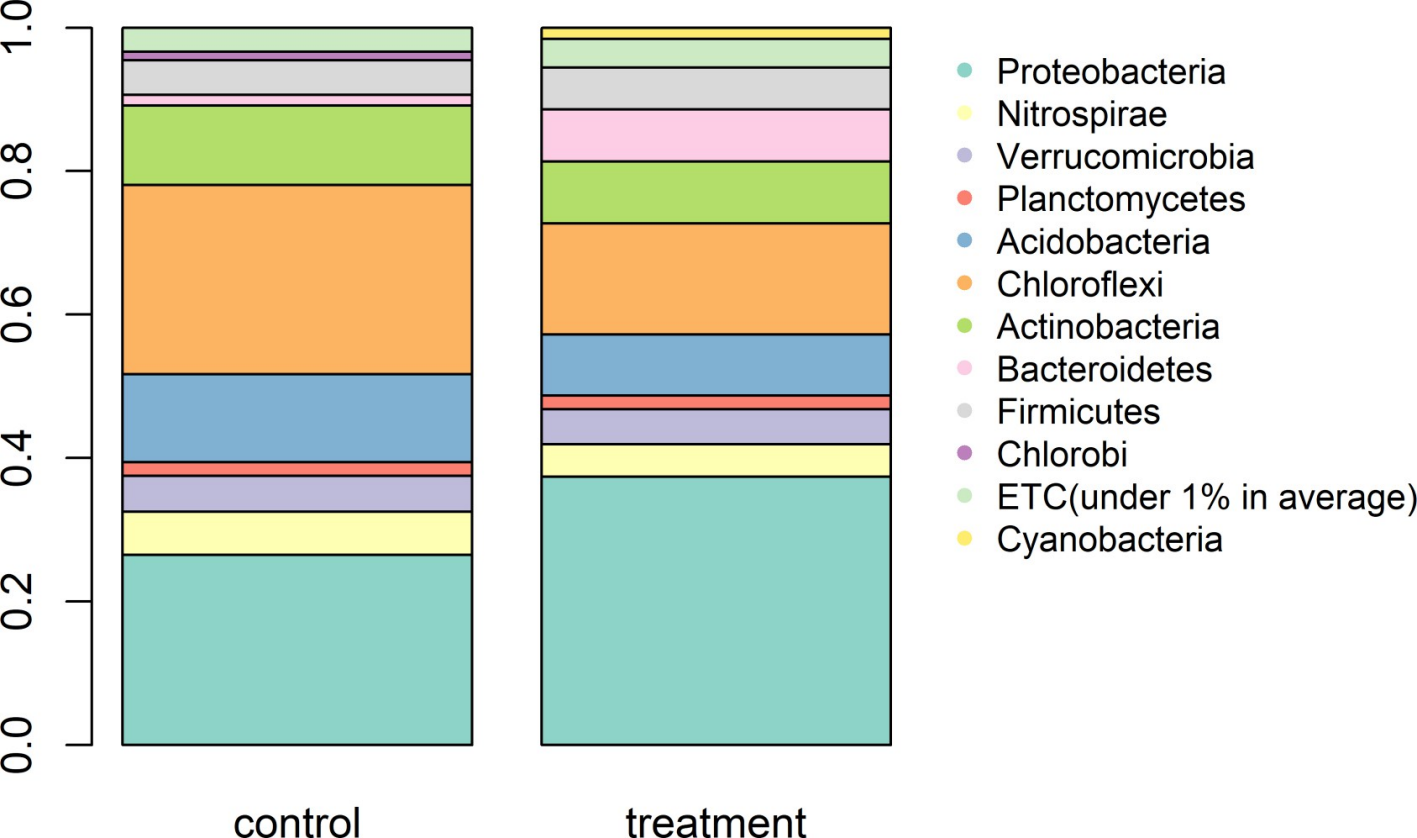
Relative abundance of bacteria at phyla level for the control and treatment soils.

Both bacterial and fungal community composition was sensitive to crane activities, and microbial community composition significantly differed between the control and treatment soils (Figs 6 and 7). For bacteria (Fig 6A), PC1 and PC2 explained 58 and 14% of the variability, respectively. Likewise, fungal communities in the treatment soils were different from those in the control soils (Fig 6B). Relative abundances of beta-proteobacteria and cyanobacteria were greater in the treatment soils, while relative abundance of chloroflexi was higher in the control soils (Fig 7). The crane migration decreased relative abundance of basidomycota, but increased relative abundance of ciliophoran (S1 Fig).

## Discussion

Here we provide evidence that the migration of cranes creates a system that differs in biogeochemical processes and biodiversity from surrounding environments. Below we discuss how migratory crane activities can drive distinct ecosystem processes via recharging, redistributing, and refreshing resources during winter.

### Three months of crane activities were enough to alter resource landscape in soils

In support of our first hypothesis, crane activities created distinct resource landscape in soils during winter compared to those without crane activities. Increases in total C and N in soils (Fig 2A and 2B) could be due to the input of crane droppings rich in C and N (Table 1) or deposit of dead body. Using the numbers reported in the national census report [30] (1,126 and 5,330 individuals of *G*.*japonensis and G*.*vipio*, respectively), we estimated how much of C and N are deposited into the soil system while cranes are present in the study site. Because the dropping weight is often 3% of the body weight [8], it is estimated that 1,126 of *G*.*japonensis* (c.9-10 kg) and 5,330 of *G*.*vipio* (c. 5-6kg) [30] would deposit approximately 1,200 kg of droppings to the system [2]. Based on the C and N concentration of the dropping (Table 1), crane droppings are estimated to supply a total of 360 kg of C and 24 kg of N to the system every day. The death of cranes is relatively rare, but on average more than 4 tolls per year have been reported in the region for the last decade [2]. Dead bodies are usually cleared up from the field immediately to avoid the spread of avian influenza, but sometimes already underwent decay when local farmers detected them (personal communication with local farmers), releasing resources to the system. Similar WEOC concentrations in soils with or without crane activities suggest that either crane activities did not significantly increase WEOC input into the soil via dropping/urea or WEOC was quickly lost via microbial activities or leaching.

In addition to the changes in the resource quantity, we demonstrate that resource quality was also influenced by crane activities. DRIFT has been increasingly used in soil science to characterize soil C functional groups [42, 51–54]. Often aliphatic group (-CH) is related to plant-derived C, while carboxylate group (-COO) is related to microbially-derived C. Thus, the relative amount of CH compared to COO implies the degree to which plant C is decomposed and processed by microbes. In our study we found that the ratio of CH over COO was significantly lower in the treatment soils (Fig 2D). Cranes may have modified the quality of soil C, via directly providing C with low CH/COO into the soil system or indirectly promoting microbial decomposition of soil C. The CH/COO of crane droppings was lower than the CH/COO of soils (Table 1 and Fig 2D). This is consistent with the findings that bird droppings are partially digested [10] and that more processed C exhibits a low CH/COO ratio [42, 54]. Thus, the input of crane droppings may have reduced the CH/COO in the treatment soils, leading to an increasing trend in the CH/COO ratio from droppings to treatment soils to control soils.

Alternatively, crane activities may have enhanced microbial decomposition of C in soil, decreasing the CH/COO. We found not only greater total (Fig 3A) and metabolically active biomass (Fig 3B), but also higher respiration (Fig 4) in the treatment soils. While previous studies report contrasting effects of resource availability on soil microbial activity [17, 18, 21–23], our results indicate that increased C and N inputs under crane activities likely stimulated microbial growth and respiration. Because CH residues are relatively fresh and less protected [42, 54], microbial communities in the treatment soils may have preferentially utilized CH residues compared to COO residues to synthesize biomass and respire.

Taken together, our results indicate that the crane migration elevated otherwise low resource availability and microbial activity during winter. We do not know if seasonal pulses of resources by crane activities would last until the following plant growing season, and if enhanced microbial biomass and activity would help plants grow better. Even though it is beyond the scope of the study, some studies imply that migratory birds can help achieve sustainable agriculture. For instance, Firth et al. [10] estimated that the attraction of ducks and geese to the agricultural fields during winter can replace N fertilizer application by c. 13%.

### Soil microbial communities were more diverse under crane activities

Our second hypothesis that crane activities will enhance microbial diversity is supported in this study. There are at least three scenarios whereby the migration of cranes could increase microbial diversity. First, crane activities may have provided resources that were limiting to microbial growth. Soil microbial communities are often shaped by resource landscape and exhibit higher diversity at greater resource availability [55–57]. As such, greater inputs of C and N under crane activities (Fig 2 and Table 1) could lead to an increase in microbial diversity. Second, more diverse substrates could have been supplied to the treatment soil, fostering a wider array of microbes. Individual microbial taxa exhibits a distinct preference and utilization efficiency for different substrates [58, 59]. The functional groups of crane droppings were distinguishable from those of soils (Fig 2 and Table 1), providing chemically different substrates. Also, cranes can transfer resources across ecosystems via foraging and dropping. As an omnivore, cranes feed N-rich fish or aquatic invertebrates from streams or reservoirs and could defecate in other places. However, because we installed trail cameras near the treatment soils, we were not able to confirm if and how much cranes forage in other area during winter. Third, crane droppings themselves may carry different microbial taxa from resident soil microbial communities. Often, migratory animals facilitate parasite dispersal across habitats [60]. Thus, it is plausible that crane droppings may have carried and deposited distinct microbial community into the system.

Consistent with Fierer et al. [61], where N gradients promoted the growth of proteobacteria while suppressing the growth of acidobacteria, we observed increased proteobacteria and decreased acidobacteria and chloroflexi under crane activities. Davis et al. [62] report that acidobacteria and chloroflexi are common in slowly growing soil bacterial community. Microorganisms manage limited amount of resources and need to choose life history strategies that represent different sets of traits [63]. For example, copiotrophs inhabit resource-rich environments and reproduce fast, while oligotrophs thrive in resource-poor environment and reproduce slowly. Thus, it is likely that resource inputs by crane activities stimulate fast-growing copiotrophs that can take advantage of the pulses of resources and start growing. Likewise, decreases in the relative abundance of basidiomycota under crane activities seem to be due to the increased resource inputs. Basidiomycota play a role in decaying relatively resistant soil C residues with a greater proportion of phenolic groups and are considered as oligotroph [64]. Therefore, the decreases in basidiomycota in the treatment soils imply that resource inputs by crane activities contained little phenolic groups, but a great quantity of easily available C to other microbes.

### Changes in soils under crane activities have ecological and policy implications

For the past decades, rice fields have gained increased attention among South Korean environmental policy makers and practitioners, as viewed as a winter habitat for migratory birds, including endangered species such as cranes [65, 66]. The Ministry of Environment introduced the Biodiversity Contract Scheme (BCS) to the rice fields of Cheorwon in 2004, a form of Payment for Ecosystem Services, which aims to offset the loss of farmers created by sparing their rice fields for wintering cranes [67]. While few grow crops in winter, farmers collect and sell rice straws, and prepare their rice fields for the following year by winter ploughing and fertilizer application. With the introduction of the BCS, farmers are encouraged to suspend these conventional practices, and instead to adopt bird-friendly practices, such as flooding their rice fields after harvest, and keeping the rice straws within the rice fields. Those farmers who join the BCS receive government subsidies in return. In recognition of the ecological importance of rice fields for migratory birds, environmental policy makers have made considerable efforts to convince more farmers to join the BCS mainly for environmental considerations, if not the monetary compensation. The results of this study, however, suggest that the BCS practices could have positive impacts on agricultural soils as these practices could enhance microbial communities through crane activities. In other words, biodiversity conservation can engender a reciprocal transaction between the cranes and farmers–habitat and food provision, and improved soils in return.

For policy makers, the agricultural potential of crane activities can serve as an additional rationale to convince more farmers to join the biodiversity schemes in Cheorwon, and other rice fields used by migratory birds for wintering habitats. Farmers of Cheorwon have long been reluctant to join crane conservation. In addition to the perceived loss, they are concerned with the possibility that the presence of endangered species in the rice fields may lead to additional farming restrictions, and even the loss of their land by being designated as protected areas. The results of this study, however, suggest that inviting cranes to their rice fields can benefit not just the birds but farmers as well because the increased C and N in the soils could enhance the productivity of their rice fields. Specifically, if the reduction in the fertilizer use serves as incentive for farmers and if the incentive weighs over the costs to flood the fields and to keep rice straws, biodiversity conservation efforts could be more welcomed among local farmers. Furthermore, the reciprocity between the cranes and farmers disrupts the prevalent thinking that puts conservation and agriculture at the oppositional ends. Instead, it suggests the possibility of farmers’ living with the cranes through carefully designed conservation practices. In this regard, this study supports recent discussions of land sharing/sparing agriculture that try to find ways to reconcile agricultural land use with biodiversity conservation [68–70].

## Conclusions

The plight of the cranes caused by a series of anthropogenic intervention perhaps echoes the widespread biodiversity loss at the global scale, confirming the recent diagnosis of the Anthropocene. While scientific studies of the Anthropocene tend to focus on the effects of anthropogenic activities on the Earth, this study pays attention to the role played by nonhuman animals to intervene with the Earth’s surface. Our results demonstrate that animal migration is not a mere movement of biomass, but an ‘ecological force’ that shapes a distinct ecosystem with altered biogeochemical processes. We find that three months of crane migration was long enough to impact microbial biodiversity and soil C dynamics in agroecosystems. Crane conservation efforts in Korea have focused on the migration itself, but taking into account the ecosystem services that cranes can provide after their migration can help appeal to a broader people. The ecological benefits gained from crane activities could offer an additional rationale for expanding biodiversity protection schemes targeted at rice fields. Further studies are required to assess if changes in resource landscape and microbial activity/composition will promote plant growth, how resource landscapes will vary along with the annual cycle of crane migration, if the beneficial effects of crane activities will be proportional to the number of cranes or depend on the types of ecosystems/management history.

## Supporting information

S1 File PCR amplification. (DOCX)

S2 File Fungal community analysis. (DOCX)

S1 Fig Relative abundance of fungi at phyla level for the control and treatment soils. (DOCX)
