# Supplementary material for: Resource landscape, microbial activity, and community composition under wintering crane activities in the Demilitarized Zone, South Korea
Source: PLoS One. 2022 May 13;17(5):e0268461. doi: 10.1371/journal.pone.0268461 (PMC9106215; doi:10.1371/journal.pone.0268461)
Supplement: S1 File — (DOCX) [file pone.0268461.s001.docx]

# **S1 File. PCR amplification**

For bacterial amplification, fusion primers of 341F (5’-AATGATACGGCGACCACCGAGATCTACAC-XXXXXXXX-TCGTCGGCAGCGTC-AGATGTGTATAAGAGACAG-CCTACGGGNGGCWGCAG-3’; underlining sequence indicates the target region primer) and 805R (5’- CAAGCAGAAGACGGCATACGAGAT-XXXXXXXX-GTCTCGTGGGCTCGG-AGATGTGTATAAGAGACAG-GACTACHVGGGTATCTAATCC-3’) were used. For fungi, we used fusion primers of UTS3-Mi (5’- AATGATACGGCGACCACCGAGATCTACAC-XXXXXXXX-TCGTCGGCAGCGTC-AGATGTGTATAAGAGACAG-GCATCGATGAAGAACGCAGC -3’; underlining sequence indicates the target region primer) and ITS4-Mi (5’- CAAGCAGAAGACGGCATACGAGAT-XXXXXXXX-GTCTCGTGGGCTCGG-AGATGTGTATAAGAGACAGTCC-TCCGCTTATTGATATGC-3’). The Fusion primers are constructed in the following order which is P5 (P7) graft binding, i5 (i7) index, Nextera consensus, Sequencing adaptor, and Target region sequence.

The amplifications were carried out under the following conditions: initial denaturation at 95°C for 3 min, followed by 25 cycles of denaturation at 95°C for 30 sec, primer annealing at 55°C for 30 sec, and extension at 72°C for 30 sec, with a final elongation at 72°C for 5 min. The PCR product was confirmed by using 1% agarose gel electrophoresis and visualized under a Gel Doc system (BioRad, Hercules, CA, USA). The amplified products were purified with the CleanPCR (CleanNA). Equal concentrations of purified products were pooled together and removed short fragments (non-target products) with CleanPCR (CleanNA). The quality and product size were assessed on a Bioanalyzer 2100 (Agilent, Palo Alto, CA, USA) using a DNA 7500 chip. Mixed amplicons were pooled and the sequencing was carried out at Chunlab, Inc. (Seoul, South Korea), with Illumina MiSeq Sequencing system (Illumina, USA) according to the manufacturer’s instructions.
