# Supplementary material for: Resource landscape, microbial activity, and community composition under wintering crane activities in the Demilitarized Zone, South Korea
Source: PLoS One. 2022 May 13;17(5):e0268461. doi: 10.1371/journal.pone.0268461 (PMC9106215; doi:10.1371/journal.pone.0268461)
Supplement: S2 File — (DOCX) [file pone.0268461.s002.docx]

# **S2 File. Fungal community analysis**

For fungi, we used unite database (<https://unite.ut.ee/>). The alpha diversity indices (Shannon and OTUs) were estimated and compared between the control and treatment soils using Wilcoxon rank-sum test. We calculated UniFrac and Bray-curtis beta diversity distances and used principal coordinates analysis to visualize the sample differences.
