# Supplementary figures and images for: Resource landscape, microbial activity, and community composition under wintering crane activities in the Demilitarized Zone, South Korea
Source: PLoS One. 2022 May 13;17(5):e0268461. doi: 10.1371/journal.pone.0268461 (PMC9106215; doi:10.1371/journal.pone.0268461)

# **S1 Fig. Relative abundance of fungi at phyla level for the control and treatment soils.**


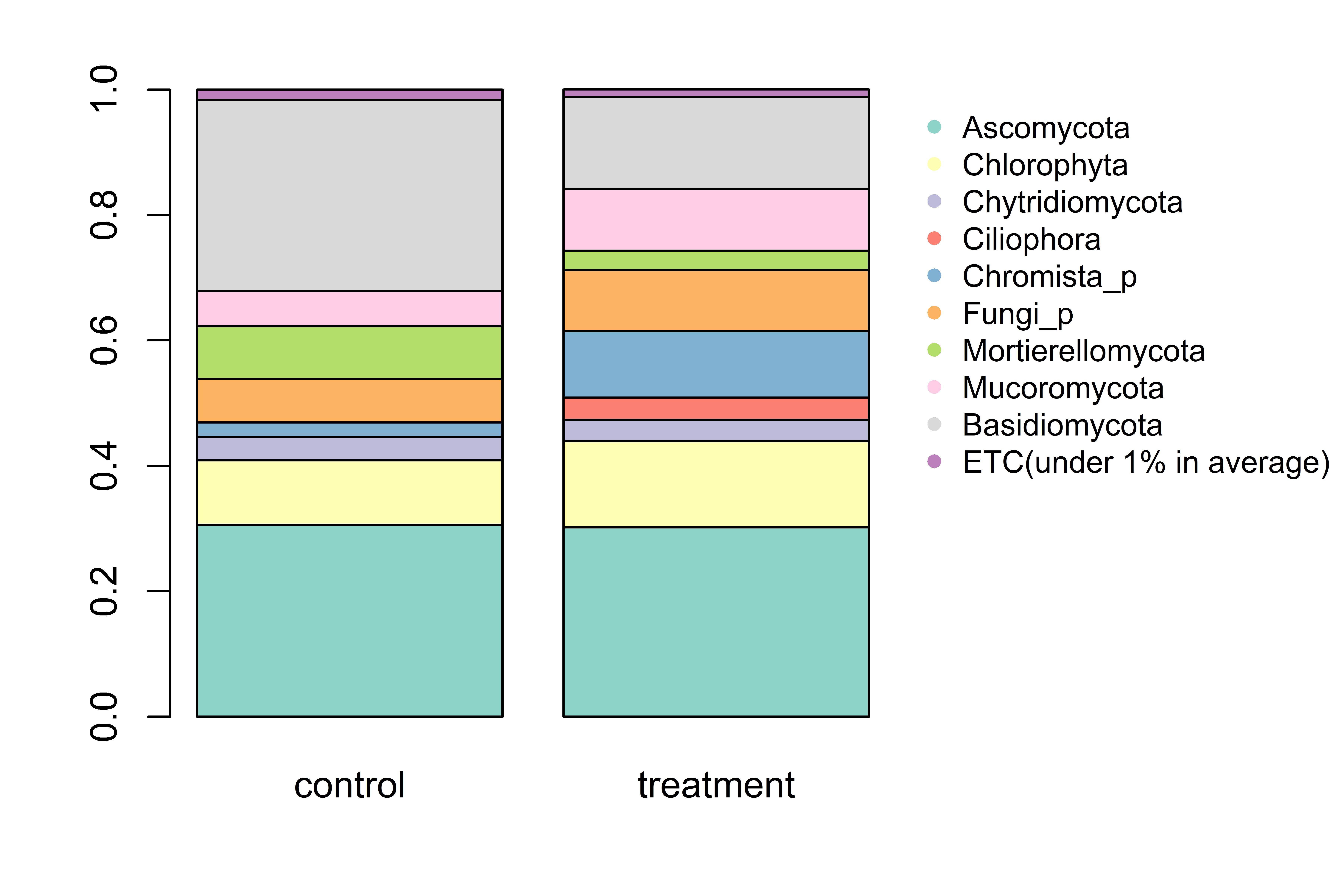

Supplement: S1 Fig — (DOCX) [file pone.0268461.s003.docx]
